# Renal sympathetic denervation versus antiarrhythmic drugs for drug-resistant hypertension and symptomatic atrial fibrillation (RSDforAF) trial: study protocol for a randomized controlled trial

**DOI:** 10.1186/1745-6215-14-168

**Published:** 2013-06-11

**Authors:** Min Qiu, Yuehui Yin, Qijun Shan

**Affiliations:** 1 Cardiovascular Department, The First Affiliated Hospital of Nanjing Medical University, No 300, Guangzhou Road, Nanjing, Jiangsu 210029, China; 2 Cardiovascular Department, The Second Affiliated Hospital of Chongqing Medical University, No 76, Linjiang Road, Chongqing, China

**Keywords:** Renal sympathetic denervation, Hypertension, Atrial fibrillation, Sympathetic nerve, Clinical trial

## Abstract

**Background:**

Recently, catheter-based renal sympathetic denervation (RSD) has been verified to be safely used to substantially reduce the levels of blood pressure, left ventricular hypertrophy, sleep apnea severity and norepinephrine spillover, and improve glucose tolerance. All these pathological changes are recognized as independent risk factors for the development and recurrence of atrial fibrillation (AF). A randomized, single-blind, parallel-control, multicenter clinical trial is being conducted to compare RSD with antiarrhythmic drugs (AAD) in patients with drug-resistant hypertension and symptomatic AF (RSDforAF trial).

**Methods/design:**

Patients with drug-resistant hypertension and symptomatic AF will be randomized to RSD and the drug treatment groups. Patients will be followed for 12 months until study closure. Up to 200 patients may be enrolled in six medical centers in China. The primary objective is to study the effects of RSD on AF burden and blood pressure in patients with hypertension and symptomatic AF.

**Discussion:**

RSDforAF trial will test the hypothesis that RSD is superior to AAD in reducing AF burden and blood pressure in patients with drug-resistant hypertension and symptomatic AF.

**Trial registration:**

ClinicalTrials.gov, NCT01713270

## Background

Atrial fibrillation (AF) is the most common arrhythmia, with the estimated prevalence of AF being 0.4 to 1% in the general population, increasing with age [1]. Hemodynamic impairment and thromboembolic events related to AF result in significant morbidity, mortality, and increase medical financial burden. So far, treatment of AF remains unsatisfactory. Pharmacological approaches for cardioversion and maintaining sinus rhythm in patients with AF appear simple but are less efficacious. The major risk related to antiarrhythmia medicine is the considerable potential drug toxicity and proarrhythmic effects. Cardiac radiofrequency catheter ablation of AF has developed rapidly in recent years, but the success rate still remains relatively low, especially in persistent AF patients. The higher recurrence of AF is also a big challenge in short- and long-term follow-up. In addition, the complications associated with AF ablation procedures are likely to result in prolonged hospitalization, long-term disability or death. Hypertension is the most important risk factor for AF, and increases the risk of thromboembolic complications [2]. In patients with AF, aggressive treatment of hypertension may retard or prevent the occurrence of AF. Recently, many clinical researchers [3-6] have verified that catheter-based renal sympathetic denervation (RSD) could be safely used to substantially reduce blood pressure, left ventricular hypertrophy and sleep apnea severity, and improve glucose tolerance. Simultaneously, a marked reduction in muscle and whole-body sympathetic nerve activity is apparent, with a decrease in renal and whole-body norepinephrine spillover. Left ventricle hypertrophy, left atrium enlargement, high norepinephrine level, glucose tolerance abnormality and obstructive sleep apnea are all recognized as independent risk factors for the development and recurrence of AF [7]. Thus, we designed this randomized, parallel-control, multicenter clinical study to demonstrate whether RSD is superior to antiarrhythmic drugs (AAD) in reducing AF burden and blood pressure in patients with drug-resistant hypertension and symptomatic AF.

## Methods

### General

The RSDforAF trial is a randomized, controlled, parallel, multicentre study. Patients with drug-resistant hypertension and symptomatic AF are randomly assigned to the medical antiarrhythmic and antihypertensive treatment group or the RSD group, with 50% of the patients assigned to each treatment arm. The first patient was randomized in July 2012. A total of 200 patients will be included and followed for a period of 12 months after inclusion. Enrollment is expected to be completed by October 2014. This study is designed to assess the safety and efficacy of RSD in patients with hypertension and symptomatic AF. The trial is registered at http://www.clinicaltrials.gov, registry number: NCT01713270. The independent medical ethics committee of the First Affiliated Hospital of Nanjing Medical University has approved this RSDforAF trial protocol, with the approval number 2012-SR-080. The institutional review board of each participating institution approved the study protocol and all the patients will provide written informed consent. All the procedures of this study are under the monitor of our hospital ethics committee.

### Patients

**Figure 1:**
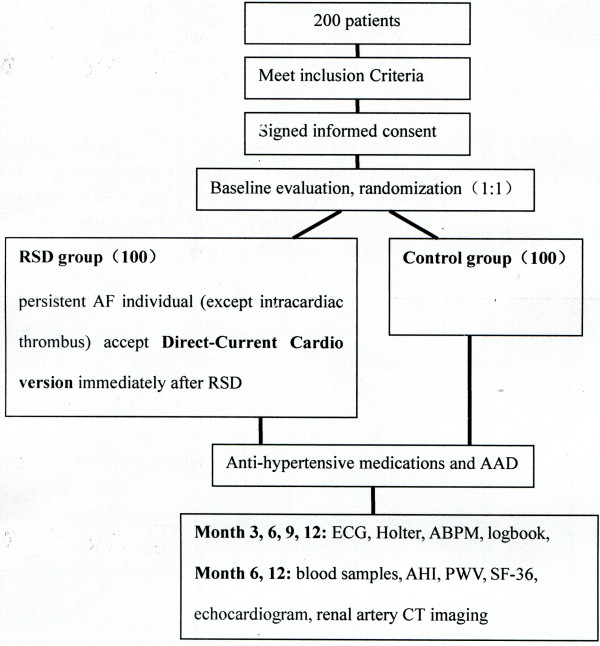
**The enrollment cascade and follow-up procedure.** AAD, Antiarrthymic drugs; ABPM, Ambulatory blood pressure monitoring; AF, Atrial fibrillation; AHI, Apnea-hypopnea index; CT, Computed tomography; ECG, Electrocardiogram; Holter; ambulatory cardiogram; PWV, Pulse wave velocity; RSD, Renal sympathetic denervation; SF-36, Short Form-36 quality-of-life questionnaire.

Adult patients who meet all inclusion/exclusion criteria (Table 1), have documented symptomatic AF (including paroxysmal and persistent AF), and have had at least one episode during the preceding 6 months are eligible for this study. Patients should be adhering to a stable antihypertensive drug regimen, including three or more antihypertensive medications of which one is a diuretic, for a minimum of 14 days prior to enrollment. If, when considering a patient’s baseline blood pressure, investigators feel that there are medication manipulations that should be attempted within 6 months, those changes should be made prior to evaluation for enrollment. If such changes are made, they must be allowed to stabilize for at least 2 weeks, and a screening blood pressure measurement must be repeated on the new medication regimen to determine if the patient qualifies for the study. As long as the participants are included, they should follow the research scheme for follow-up visits and other requirements. The enrollment cascade and follow-up procedure are presented in Figure 1.

**Table 1:** Study inclusion/exclusion criteria

**Inclusion criteria**	**Exclusion criteria**
1 Individual is ≥18 and ≤75 years of age	1 Secondary and white-coat hypertension
2 More than 6 months for definite primary hypertension	2 Permanent AF individual
3 Individual has a systolic blood pressure ≥160 mmHg (≥150 mmHg for type 2 diabetics) based on an average of three office blood pressure readings measured	3 Thrombus in left atrial appendage found by transesophageal echocardiography
4 Individual is adhering to a stable drug regimen, including three or more antihypertensive medications of which one is a diuretic, for a minimum of 14 days prior to enrollment	4 Individual with severely enlarged left atria ≥55 mm
5 At least 30 seconds on a rhythm strip in an ECG record and at least 1 AF outbreak which was recorded by EGG and Holter during the preceding 6 months	5 Individual has experienced renal artery stenosis, or a history of prior renal artery intervention including balloon angioplasty or stenting, or ineligible conditions seen on renal artery computed tomography angiogram inspection such as double renal artery on one side, renal artery length ≤2 cm, diameter ≤4 mm, and distortion at incept sect
6 Paroxysmal and persistent AF individual	6 Individual has experienced a definite acute coronary syndrome in the past 3 months, or a cerebrovascular accident and alimentary canal bleeding within 3 months
7 Agree to attend experimental clinic and sign written informed consent	7 Individual has experienced sick sinus syndrome
	8 reversible causes of AF, including alcohol abuse, surgery, electrocution, myocadial infarction, pericarditis, myocarditis, pulmonary embolism or other pulmonary diseases, hyperthyroidism, and other metabolic disorders
	9 structural heart diseases such as congenital, valvular heart diseases and kinds of cardiomyopathy
	10 Individual is pregnant or nursing
	11 Mental disorders - individual cannot complete follow-up or one the researcher thinks is unfit to be included in this study
	AF, atrial fibrillation; ECG, electrocardiogram.

### Baseline evaluation and randomization

Prior to enrollment, patients will provide signed informed consent and undergo a full baseline evaluation, including physical examination, medical history, medicine, New York Heart Association functional class, 12-lead electrocardiogram, 24-hour ambulatory electrocardiogram (Holter), 24-hour ambulatory blood pressure monitoring, apnea-hypopnea index, pulse wave velocity, quality of life (using Short Form-36 quality-of-life questionnaire), echocardiogram, renal artery CT imaging, transesophageal echocardiography (only persistent AF patients), and blood testing (serum creatinine, blood lipid, glycosylated hemoglobin, fasting blood glucose). The echocardiogram is performed to measure left ventricular ejection fraction, left ventricular end-diastolic diameter, interventricular septum and left atrium diameter. All the included patients should have no severe mitral valve insufficiency, and no very large left atrium (≥55 mm). Renal artery CT imaging scans the suitability of the anatomy for treatment - anatomic eligibility is defined as bilateral single main renal arteries >4 mm in diameter and >20 mm in length, without significant stenosis or other abnormality.

Patients meeting all selection criteria, and having anatomic eligibility documented by renal artery imaging will be randomized. Randomization will be stratified by study center and will be at a 1:1 ratio for the RSD group to the drug treatment group. To obtain nonbiased information and perform nonbiased assessments, clinicians and their staff who collect and assess system data are blinded to the treatment. Unblinded clinicians and staff will be responsible for randomization, intervention processes, and monitoring system performance. Using the intention-to-treat analysis method of this study, these patients will be followed in their original randomization assignment.

### Intervention

#### Renal sympathetic denervation group

The technique of renal denervation has recently been described by Ahmed and colleagues [8]. After standard femoral vascular access, contrast renal angiography was performed to localize and assess the renal arteries for accessibility and appropriateness for RSD. Once the anatomy was deemed acceptable, the internally irrigated radiofrequency ablation catheter (Celcius Thermocool, Biosense Webster, Diamond Bar, CA, USA) was introduced into each renal artery. This was then maneuvered within the renal artery to allow energy delivery in a circumferential, longitudinally staggered manner to minimize the chance of renal artery stenosis. About four to eight ablations at 10 W for 60 seconds each were performed in both renal arteries. During ablation, the catheter system monitored tip temperature and impedance, altering radiofrequency energy delivery in response to a predetermined algorithm. Visceral pain at the time of energy delivery was managed with 0.05 to 0.1 mg intravenous fentanyl. After renal sympathetic denervation, patients with persistent AF accepted direct-current cardioversion immediately.

If clinically indicated, class IC AAD treatment (propafenone) is a first-line therapy for patients without contraindications, otherwise amiodarone is the drug of choice, and also metoprolol may be prescribed. If necessary, AAD can be supplemented by agents such as digoxin, diltiazem and metoprolol that slow the atrioventricular conduction. Combination of several AAD is not allowed. Recommended drugs are shown in Table 2. After 3 months, AAD treatment is discontinued. Efforts will be made to document any relapse of symptomatic arrhythmia by either 12-lead electrocardiogram or Holter monitoring. The use of AAD treatment is monitored closely throughout the study period in both treatment arms.

**Table 2:** Recommended antiarrhythmic drugs in the study

**1st choice**	**2nd choice**	**3rd choice**
Propafenone	Amiodarone	Metoprolol

Antihypertensive medications are intended to be maintained at baseline doses (in both RSD and the drug treatment group) for at least 6 months to evaluate the primary endpoint of effectiveness of RSD without medication changes confounding the results. However, in cases where medication changes are considered medically necessary (for example, significant blood pressure lowering or adverse events directly related to blood pressure or blood pressure medications), alteration may be made as necessary.

#### Drug treatment group

Patients in the drug treatment group will be followed-up at 3, 6, 9 and 12 months after randomization. All the patients in this group will take their baseline antihypertensive medication at the original doses, without any changes except when medically required. AAD treatment is consistent in both arms.

### Antithrombotic strategies

Oral anticoagulation (warfarin, INR 2.0 to 3.0) is recommended for at least 2 months in both arms [7]. After direct-current cardioversion fails in patients with persistent AF, anticoagulation therapy is continued throughout the study period to obtain a stable INR of 2.0 to 3.0.

### Follow-up and data management

Prior to discharge, the research staff shall review study requirements with the patient to ensure compliance with the follow-up schedule. Figure 1 lists the work-ups and procedures required at each follow-up time for the drug treatment and RSD groups.

Individual patient log-books are filled in with the following data: (i) daily home blood pressure, heart rate and medications (at least twice a week), (ii) episodes of symptomatic arrhythmia, and (iii) cause of changing or stopping medicine.

All study data will be recorded onto case report forms. The principal investigator remains responsible for the accuracy and integrity of all data entered on case report forms.

### Primary endpoints

The primary endpoint of this study is to demonstrate the effect of RSD on AF burden in patients with hypertension and symptomatic AF.

### Secondary endpoints

The secondary endpoints consist of the change in office systolic blood pressure from baseline to 12 months post-randomization, changes in cardiac structure and function by echocardiogram (include left ventricular ejection fraction, left ventricular end-diastolic diameter, interventricular septum, left atrium diameter), autonomic nerve function (heart rate variability by Holter), fasting blood glucose, glycated hemoglobin, blood lipid, apnea-hypopnea index, pulse wave velocity and quality of life.

### Sample size and statistical analysis

It is assumed that 27.75% of AAD-treated patients [7] and 40% of RSD patients [9] will be AF-free after 1 year, and the two groups obey a binomial distribution and have the same variance, each treatment arm including 76 patients. By two-tailed binomial tests, the difference between the two groups can be detected with a power of 90%. To account for an approximate 20% premature withdrawal rate prior to the 12-month time point, 180 patients will be randomized. As discussed above, it is expected that approximately 200 patients will be enrolled into the baseline phase in order to achieve 180 randomized patients. All analyses will be carried out according to the intention-to-treat-principle.

## Adverse events

This is a fairly new technique and the information available so far indicates it is very safe. No death has occurred and there has been no documented lasting damage to a kidney. The most common side effect we have observed is bruising at the groin. Other rare but potentially more serious side effects that have been described are as follows:

1. Acute renal artery dissection. This can usually be dealt with by placing a stent in the damaged artery.

2. Persistent abdominal pain lasting beyond the time of the procedure.

3. Low heart rate.

4. Large drop in blood pressure. This may require patients to stay in hospital for a few days to adjust their medication.

5. Damage to the artery in the groin. This occurs in about 1% of cases. Blood transfusion or rarely a small procedure or operation to the artery may be required.

6. Risk of failure to respond to renal sympathetic denervation in 10 to 15% of cases.

Other possible side effects that have not been seen with RSD, but which can occur after any procedure where an artery is catheterized include an allergic reaction to the X‒ray contrast, or contrast-induced nephropathy.

## Discussion

Cardiac catheter ablation for AF has become a very commonly and widely accepted procedure in recent years, but the low success rate and high recurrence after procedure in the short and long term is disappointing. A recent report of the long-term success rate in patients with symptomatic drug-refractory AF ablated at a prestigious institution was dismal at 23% after 6 years of follow-up [10]. When Cosedis and colleagues [11] compared radiofrequency catheter ablation with AAD therapy as first-line treatment in patients with paroxysmal AF, there was no significant difference between the ablation and drug therapy groups in the cumulative burden of AF over a period of 2 years. Furthermore, left atrial ablation probably leads to serious complications, including cardiac perforation, stroke, pulmonary vein stenosis, and atrial esophageal fistula formation.

So far, the most common risk factor of AF is hypertension. Research on Chinese hospitalized patients revealed that more than half of AF sufferers had the co-morbidity of hypertension. Hypertension is associated with left ventricular hypertrophy, impaired ventricular filling, left atrial pressure increases, left atrial enlargement, and slowing of atrial electrical conduction velocity. These changes in cardiac structure and electrical characters, also called remodeling, favor the development and maintenance of AF. AF itself increases the risk of thromboembolic complications [2]. Based on 38-year follow-up data from the Framingham Study, echocardiographic predictors of AF include left atrial enlargement (39% increase in risk per 5 mm increment) and left ventricular wall thickness (28% per 4 mm increment) [12]. Another study examined the impact of increasing atrial pressure on pulmonary vein muscle sleeve activation, and found that, as left atrial pressure was increased above 10 cmH_2_O, the left atrial–pulmonary vein junction became the source of dominant rotors [13]. In addition, the renin-angiotensin-aldosterone system activity increased in hypertensive heart disease. Angiotensin II is a well characterized profibrotic molecule, giving rise to atrial fibrosis and conduction heterogeneity [14]. Lau and colleagues [15] have confirmed that hypertension is associated with early and progressive changes in atrial remodeling. Atrial remodeling occurs at different time domains in chronic hypertension with significant electro-structural correlation of the remodeling cascade. Antihypertensive treatment at an early stage may prevent formation of a substrate capable of maintaining AF.

Sympathetic nerve activity is crucial for initiation and maintenance of systemic hypertension. It plays an important role in the development and maintenance of AF. Animal models indicated that beta-adrenergic stimulators agents (isoproterenol and adrenaline) can increase the occurrence rate of AF by programmed electrical stimulation [16,17]. In a study by Oraland and colleagues [18], among the 80 patients with paroxysmal AF, persistent AF was induced in 67 patients (84%) by isoproterenol. Recently, clinical studies have verified that metoprolol was effective in preventing relapse into AF or atrial flutter after cardioversion of persistent AF [19,20]. Both local and international scholars found that significant nerve sprouting and sympathetic hyperinnervation were present in animal models of sustained AF produced by prolonged right atrial pacing. Over-activity and inhomogeneous distribution of regional sympathetic nerve play an important role in AF induction [21,22].

Sympathetic activation of the heart increases calcium entry and the spontaneous release of calcium from the sarcoplasmic reticulum, and thus shortens the action potential duration and atrial refractory period. Abnormal intracellular calcium handling and short action potential durations are key features of electrophysiological remodeling in AF [23]. In 2012, using animal models, Zhao and colleagues [24] reported that episodes of AF could be decreased by renal sympathetic denervation during short-term rapid atrial pacing. This effect might have a relationship with the decreased activity of the renin-angiotensin-aldosterone system. In a swine model for obstructive sleep apnea, renal denervation displayed antiarrhythmic effects by attenuation of negative-tracheal-pressure-induced atrial effective refractory period shortening [25]. Therefore, AF inducibility was reduced, and simultaneously, postapneic blood pressure rises were inhibited. In another study with 12 swine undergoing renal denervation [26], heart rate as well as atrioventricular nodal conduction was reduced during sinus rhythm. After 30 minutes of atrial tachypacing, the duration of induced AF episodes was significantly shortened compared with sham controls . Accordingly, we hypothesize that renal sympathetic denervation could have a salutary effect on AF patterns in patients with hypertension by improving blood pressure control and by reducing central sympathetic cardiac driving.

Renal sympathetic denervation is a newly available therapeutic option for drug-resistant hypertension. In 2009, the Australian researchers Krum and colleagues [3] reported for the first time that percutaneous renal sympathetic denervation acquired positive and sustained blood pressure reduction in patients with resistant hypertension. Post-procedure office systolic/diastolic pressures were reduced by 32/14 mmHg at 24 months, without serious adverse events. Later, Witkowski and colleagues [4] studied 10 patients with refractory hypertension and sleep apnea who underwent renal denervation and completed 6-month follow-up evaluations. Significant decreases in office systolic and diastolic blood pressures were observed; plasma glucose concentration 2 hours after glucose administration and the hemoglobin A1C level, as well as the apnea-hypopnea index at 6 months, were also decreased remarkably. In addition to the known effect on blood pressure, the study by Brandt and colleagues [5] showed renal denervation significantly reduces left ventricular mass and improves diastolic function. In another study, there was a marked reduction in muscle and whole-body sympathetic nerve activity, with a decrease in renal and whole-body norepinephrine spillover [6]. Left ventricle hypertrophy, left atrial enlargement, high norepinephrine levels, glucose tolerance abnormality and obstructive sleep apnea are all recognized as independent risk factors for the development and recurrence of AF. Recently, a case was reported with renal artery ablation instead of pulmonary vein ablation in an uncontrolled hypertensive patient with symptomatic, drug-resistant persistent AF [27]. Six months later, the patient presented marked improvement in symptoms and exercise capacity, and was still free of symptoms and in normal sinus rhythm; echocardiography showed a progressive decrease of the left atrial diameter from 45 mm just prior to renal ablation to 40 and 36 mm at 3 and 6 months thereafter, respectively. Pokushalov and colleagues [9] found that, at 1 year follow-up, the success rate (that is, being AF free) for the combined procedure was achieved in 9 of the 13 patients (69%) treated with pulmonary vein isolation and renal artery denervation as compared with only 4 of 14 patients (29%) in those treated with only pulmonary vein isolation.

Should renal artery denervation be part of the ablation procedure for all patients undergoing catheter ablation for the treatment of AF, or should renal artery denervation be performed instead of left atrial ablation? Larger clinical studies are needed to help answer these questions. The RSDforAF trial is testing the hypothesis that RSD is superior to AAD in reducing AF burden and blood pressure in patients with drug-resistant hypertension and symptomatic AF.

## Trial status

The trial is registered at http://www.clinicaltrials.gov, registry number: NCT01713270. The first patient was randomized in July 2012. This study is currently recruiting participants.

## Abbreviations

AAD: Antiarrhythmic drugs; AF: Atrial fibrillation; CT: Computed tomography; RSD: Renal sympathetic denervation.

## Competing interests

The authors declare that they have no competing interests.

## Authors’ contributions

QS contributed to the design and conception of this study, provided critical revision of the article, and approval of article. YY contributed to the design and conception of this study. MQ drafted the manuscript. All authors read and approved the final manuscript.
